# Enhancing immune response and survival in hepatocellular carcinoma with novel oncolytic Jurona virus and immune checkpoint blockade

**DOI:** 10.1016/j.omton.2024.200913

**Published:** 2024-11-26

**Authors:** Mulu Z. Tesfay, Yuguo Zhang, Khandoker U. Ferdous, Mika A. Taylor, Aleksandra Cios, Randal S. Shelton, Camila C. Simoes, Chelsae R. Watters, Oumar Barro, Natalie M. Elliott, Bahaa Mustafa, Jean Christopher Chamcheu, Alicia L. Graham, Charity L. Washam, Duah Alkam, Allen Gies, Stephanie D. Byrum, Emmanouil Giorgakis, Steven R. Post, Thomas Kelly, Jun Ying, Omeed Moaven, Chiswili Y. Chabu, Martin E. Fernandez-Zapico, Dan G. Duda, Lewis R. Roberts, Rang Govindarajan, Mitesh J. Borad, Martin J. Cannon, Alexei G. Basnakian, Bolni M. Nagalo

**Affiliations:** 1 Department of Pathology, University of Arkansas for Medical Sciences (UAMS), Little Rock, AR, USA; 2 The Winthrop P. Rockefeller Cancer Institute, UAMS, Little Rock, AR, USA; 3 Department of Pharmacology, UAMS, Little Rock, AR, USA; 4 Department of Microbiology and Immunology, UAMS, Little Rock, AR, USA; 5 Department of Molecular Medicine, Mayo Clinic, Rochester, MN, USA; 6 School of Basic Pharmaceutical and Toxicological Sciences, College of Pharmacy, University of Louisiana Monroe, Monroe, LA, USA; 7 Department of Biostatistics, UAMS College of Public Health, Little Rock, AR, USA; 8 Edwin L. Steele Laboratories for Tumor Biology, Department of Radiation Oncology, Massachusetts General Hospital, Boston, MA, USA; 9 Schulze Center for Novel Therapeutics, Division of Oncology Research, Mayo Clinic, Rochester, MN, USA; 10 College of Medicine, Surgery Transplant University of Arkansas for Medical Sciences (UAMS), Little Rock, AR, USA; 11 Division of Surgical Oncology, Department of Surgery, Louisiana State University (LSU) Health, New Orleans, LA, USA; 12 Department of Interdisciplinary Oncology, Louisiana Cancer Research Center, Louisiana State University (LSU) Health, New Orleans, LA, USA; 13 LSU-LCMC Cancer Center, New Orleans, LA, USA; 14 Division of Biological Sciences, University of Missouri, Columbia, MO, USA; 15 Department of Surgery, School of Medicine, University of Missouri, Columbia, MO, USA; 16 Siteman Cancer Center, Washington University, St. Louis, MO, USA; 17 Department of Pharmaceutical Sciences, College of Pharmacy, University of Arkansas for Medical Sciences (UAMS), Little Rock, AR, USA; 18 Medical Oncology Division, Internal Medicine Department, The University of Arkansas for Medical Sciences, Little Rock, AR, USA

**Keywords:** *Rhabdoviridae*, vesiculovirus, Jurona virus, oncolytic virus, hepatocellular carcinoma

## Abstract

Members of the *Vesiculovirus* genus including Jurona virus (JURV) have emerged as promising immunotherapeutic agents, characterized by their tumor selectivity, fast kinetics, low seroprevalence, and minimal toxicity in humans. Here, we demonstrate that the administration of JURV leads to tumor regression in both hepatocellular carcinoma (HCC) xenograft and syngeneic models. Furthermore, our findings indicate that combining JURV and anti-PD-1 therapy reduced tumor burden and improved survival rates over JURV or anti-PD-1 alone in an orthotopic HCC model. Proteogenomic analysis of JURV-treated, murine HCC tumors demonstrates that the therapeutic effects of the combination of JURV and anti-PD-1 are predominantly driven by coordinated activation of immune effectors, which modulate the tumor microenvironment into a state conducive to anti-tumor activity. Our results establish JURV as a potent candidate for immunovirotherapy in HCC, capable of modulating immune response and synergizing with standard of care for HCC to prolong survival in preclinical models. Further, this research deepens our understanding of JURV’s anti-tumoral mechanisms and highlights its potential as a novel approach to HCC treatment strategies.

## Graphical abstract

**Figure:**
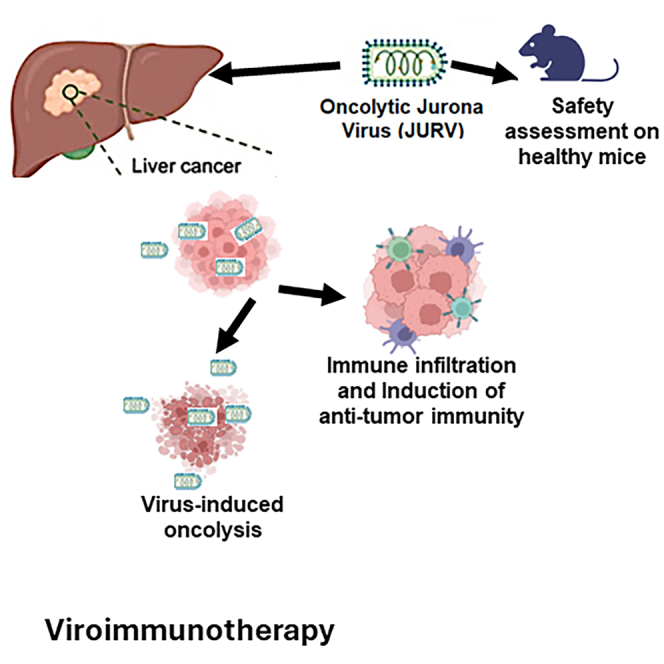

## Teaser

Tesfay and colleagues, using both *in vitro* and preclinical models of hepatocellular carcinoma, show that the administration of Jurona virus, a non-pathogenic oncolytic virus, effectively induces tumor cell death and increases T cell infiltration. Treatment with Jurona virus and anti-PD1 antibody significantly extends survival in hepatocellular carcinoma tumor models.

## Introduction

The incidence of hepatocellular carcinoma (HCC), the predominant form of primary liver cancer, is increasing more rapidly than that of any other cancer in the United States.^1^ HCC is frequently diagnosed at advanced stages, making surgical resection or liver transplantation for curative purposes challenging.^2^^,^^3^

Recent advancements in first-line treatments for advanced HCC involve combination therapies that include immune checkpoint blockade (ICB), specifically anti-programmed cell death protein/ligand 1 (PD-1/PD-L1) antibodies, combined with anti-angiogenics.^4^ Despite these advances, the response rates to these therapies hover around 15%–20%, with median survival for patients with advanced unresectable HCC ranging from 1 to 2 years.^5^

For patients who respond, tumor-infiltrating lymphocytes (TILs) correlate strongly with better outcomes. Patients with high TILs in their tumors typically experience better responses to the combination of ICB and anti-angiogenic therapy, leading to increased overall survival rates.^6^^,^^7^^,^^8^^,^^9^ However, HCC tumors often lack tumor-associated antigens that are strongly and consistently expressed and capable of triggering anti-tumor immune responses or being targeted through adoptive immunotherapy or vaccination. In addition, the presence of cells that suppress T cell responses in the tumor microenvironment (TME) further limit immune response against tumors.^6^ In this context, developing strategies to increase immune responses within HCC tumors may open new opportunities for maximizing therapeutic potential for patients with advanced HCC.

Oncolytic viruses (OVs) have emerged as a promising strategy to enhance the immunogenicity of tumors by directly lysing cancer cells and inducing an immune response.^10^^,^^11^ OVs promote tumor cell death and increase the recruitment and activation of TILs within the TME. This dual action can potentiate the effects of standard therapies, such as ICB and anti-angiogenic agents, by modifying the immune landscape of the tumor and making it more receptive to treatment.^10^^,^^11^ Among the attractive class of OVs, the *Rhabdoviridae* family, specifically the *vesiculovirus* genus, has garnered significant interest due to their inherent advantages over other viral vectors, including board tropism, fast replication in cancer cells, cytoplasmic replication, genetic manipulability, and low human seroprevalence.^11^

Here, we show that Jurona virus (JURV),^12^ a member of the *vesiculovirus* genus, effectively induces cytolytic activity in HCC cells *in vitro* and delays tumor progression *in vivo*. Moreover, we demonstrate that JURV is safe and elicits systemic anti-tumor immunity, inhibiting growth in both virus injected and distal tumors in a syngeneic HCC model. Furthermore, administration of JURV remodeled the TME by enhancing the activation of tumor-specific cytotoxic T cells and, when combined with ICB, improved survival in an aggressive orthotopic murine model of HCC. These results lay a foundational basis for further exploration of JURV and the combination of JURV with ICB as a novel therapeutic approach in HCC treatment.

## Results

### Rescue of JURV

We obtained JURV from the University of Texas Medical Branch World Reference Center for Emerging Viruses and Arboviruses (Galveston, TX). It has been isolated from *Haemagogus* sp. and a human in northern Brazil.^12^ A laboratory-adapted viral clone of JURV was generated using sequential plaque purifications in Vero cells (ATCC, cat. no. CCL-81; RRID: CVCL_0059). RNA sequencing was applied to confirm the full-length JURV genome (10,993 bp) as described previously.^13^ Analysis of the genome of JURV showed an identical genome organization as that observed in *Vesicular stomatitis virus* (VSV) and *Morreton virus* (MORV) (Figures S1A–S1C), two other members of the *Rhabdoviridae* family.^5^ Infectious JURV was recovered from a full-length cDNA clone (GenScript) comprising genes encoding for the nucleoprotein (JURV-N), phosphoprotein (JURV-P), matrix protein (JURV-M), glycoprotein (JURV-G), and RNA-directed RNA polymerase L protein (JURV-L), as described in the materials and methods.

### *In vitro* cytotoxicity activity of JURV in HCC cells

**Figure 1:**
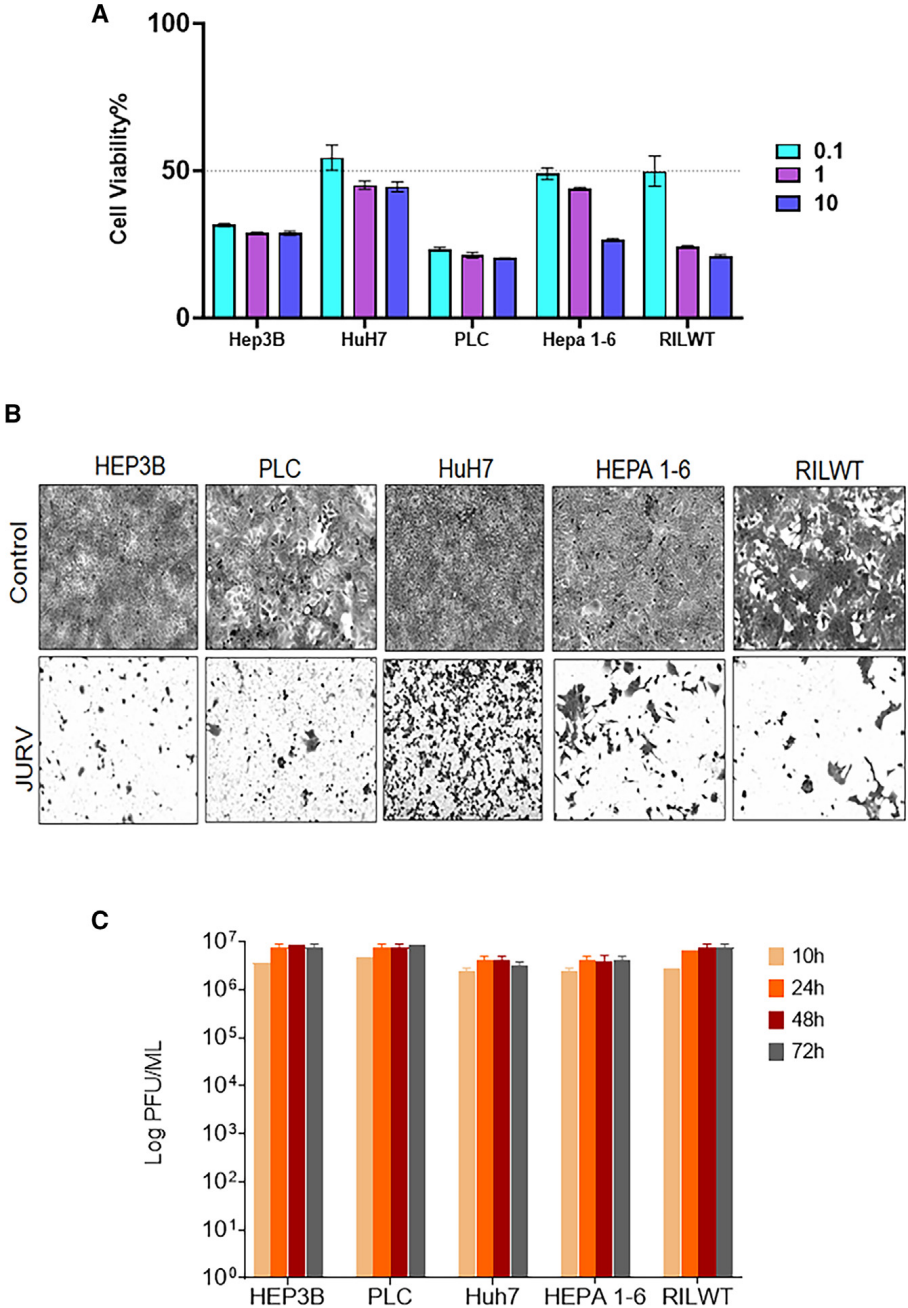
Oncolytic JURV is effective at inducing oncolysis in HCC cell lines (A) Monolayers of human HCC (HEP3B, PLC, HuH7), murine HCC (HEPA 1–6 and RILWT) were seeded at a density of 1.5 × 10^4^/well in 96-well plates and infected with JURV at an MOIs of 10, 1, or 0.1, respectively. The percentage of cell viability was determined 72 h post-infection using a colorimetric assay (MTS, Promega) and calculated as percent of noninfected control cells. The discontinued lines on the graphs indicate the cutoff percentage for resistance (>50% cell viability above the line) and sensitivity (<50% of cell viability, below the line). Data were collected from multiple replicates over three independent experiments. Bars indicate mean ± SEM. (B) Crystal violet staining. Cancer cells were plated at 5.0 × 10^5^/well in a 6-well plate and rested overnight. The following day they were infected with JURV at an MOI of 0.1. Cells were fixed and stained with crystal violet 72 h post-infection, and images were captured at 10× magnification on an Olympus IX83 Inverted Microscope System. (C) HCC cells were plated in 6-well plates at 2.0 × 10^5^/well and infected with JURV at an MOI of 0.1. Supernatants from infected cells were collected at different time points, and viral titer was determined using a TCID_50_ (50% tissue culture infective dose) or PFU method on Vero cells (1.5 × 10^4^). Data are plotted from two independent assessments of TCID_50_ for each point with mean ± SEM.

We assessed the *in vitro* cytotoxicity of JURV in various human and murine HCC lines, including HEP3B, PLC, HuH7, HEPA 1–6, and RILWT. These cell lines were infected with JURV at multiplicities of infection (MOIs) of 0.1, 1, and 10 (Figure 1A). With an MTS cell viability assay at 72 h post-infection, we observed a reduction in cell viability across all cell lines, with differences in response to each cell type. HEP3B and PLC cells showed a ∼30% reduction in cell viability irrespective of the MOI (Figure 1A), while the other cell lines showed MOI-dependent cell cytotoxic effects, mostly reaching ∼30% at high MOI. Crystal violet staining was performed 3 days post-infection with an MOI of 0.1. It showed that JURV infection resulted in the substantial loss of adherent cells in most cell lines, except HuH7 (Figure 1B), indicating that the MTS assay might have underestimated JURV’s oncolytic impact. In addition, the viral kinetic analysis revealed that JURV amplification reached around 10^6^ plaque-forming units (PFU)/mL viral titers in HCC cell supernatants as early as 10 h post-infection (Figure 1C), indicating JURV’s high infectivity and fast replication capability in these HCC cells.

### *In vivo* safety assessment of JURV in non-tumor-bearing mice

**Figure 2:**
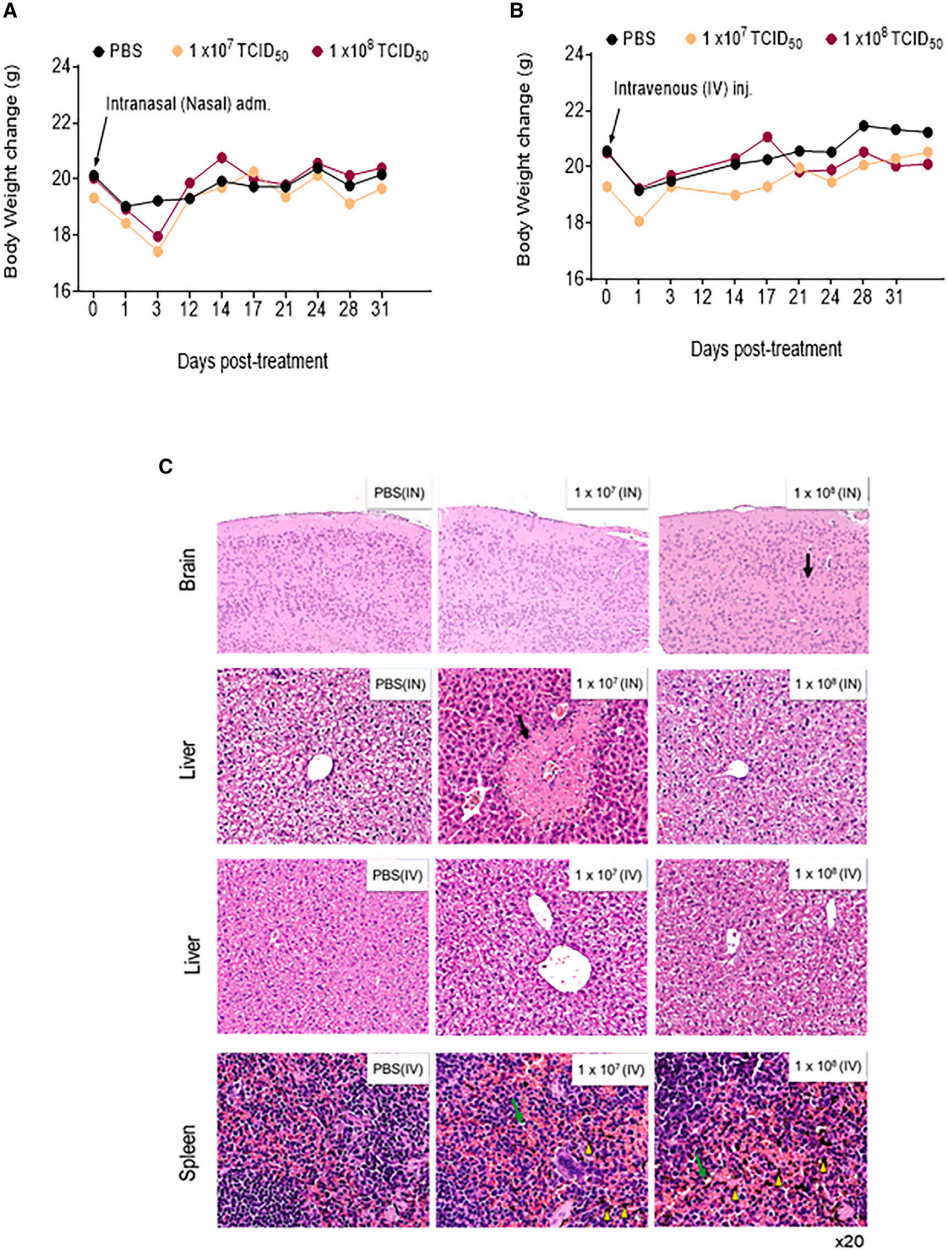
Effects of low and high doses of oncolytic JURV on body weight and hemogram in mice Non-tumor-bearing female C57BL6/J (*n* = 6/group; strain no. 000664) of age 6–8 weeks were administered single doses of PBS, 1 × 10^7^ TCID_50_ of JURV, or 1 × 10^8^ TCID_50_ of JURV (A) intranasally (i.n.) or (B) intravenously (i.v.). Body weight was recorded twice a week in both the i.n. and i.v. cohorts to assess drug-related toxicity. Three mice per group in each cohort (i.n. or i.v.) were sacrificed 3 days post-infection, and blood, brain, and liver were harvested to assess the short-term toxicity. Hematoxylin and eosin (H&E) staining (brain, spleen, and liver) are shown for i.n. and i.v. administration (C), where black arrows indicate that samples were within normal limits. Green arrows indicate necrosis, single cell, macrophage, sporadic. Yellow triangles indicate pigmentation increased in macrophages, red pulp, and white pulp.

We assessed the safety profile of JURV using two doses (1.0 × 10^7^ or 1.0 × 10^8^ TCID_50_ [50% tissue culture infective dose]) of JURV that are around 5- to 50-fold higher than the toxic threshold for VSV (1 × 10^6^ PFU).^14^ Doses were administrated to non-tumor-bearing healthy mice either intranasally (i.n.) or intravenously (i.v.). Our analyses, including post-infection body weight monitoring and histological examination of key organs (brain, liver, or spleen), revealed a mild weight loss (10%–15%) in the initial 3 days but no significant histopathological changes in the brain, liver, or spleen (Figures 2A–2C). Importantly, there were no marked differences in clinical signs such as paralysis, death, fur condition, or serum markers of drug-induced toxicity (Figures S4A–S4O) between the JURV-treated and control groups, indicating that high-dose JURV administration is not associated with severe adverse effects in this model.

### Intratumoral administration of JURV demonstrates significant anti-tumor activity in HEP3B-xenograft HCC models

**Figure 3:**
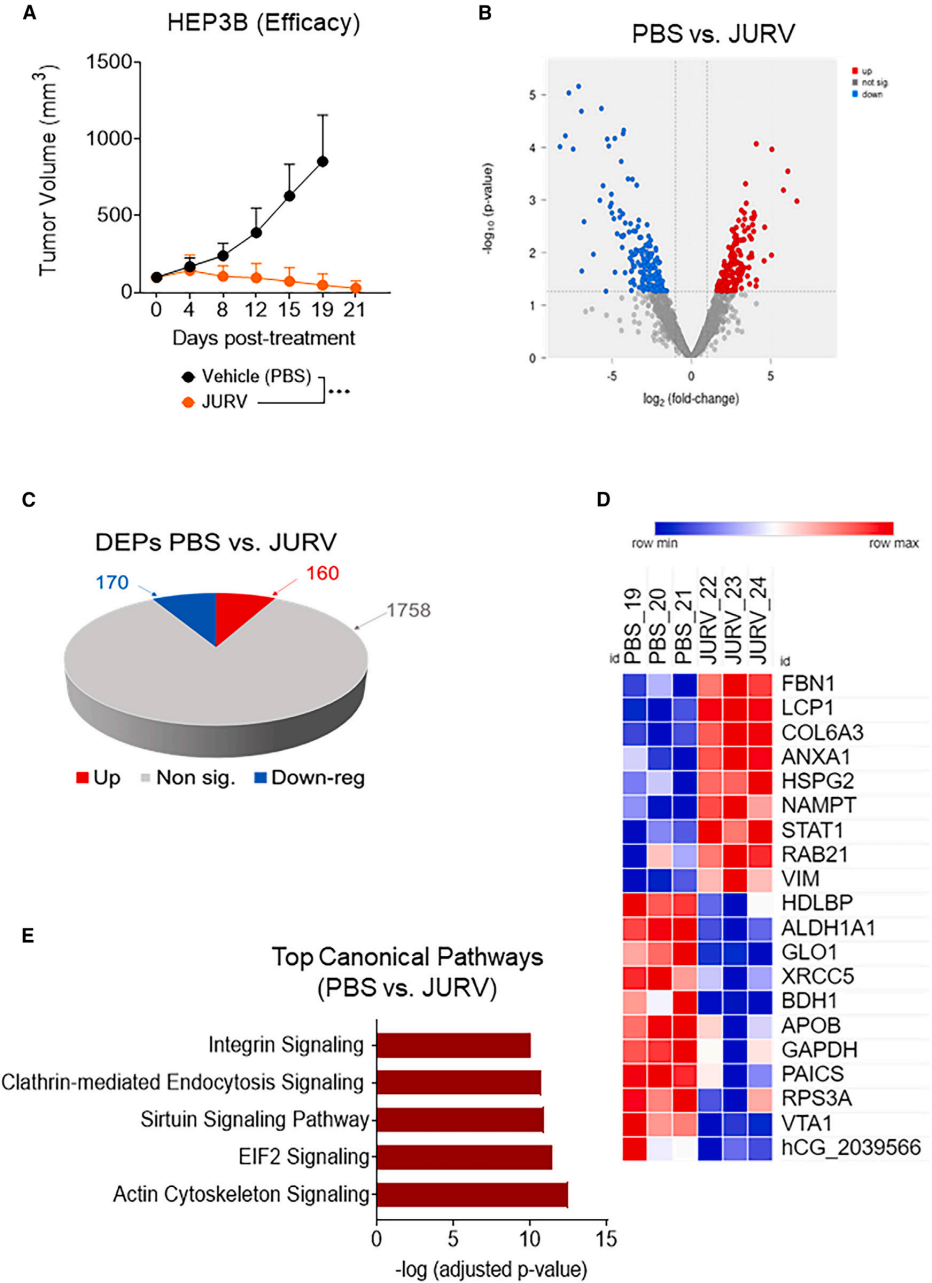
Assessment of JURV-mediated oncolysis in Hep3B xenografts Female NOD.Cg-Prkdcscid/J (strain no. 001.0303) mice (*n* = 6/group) were inoculated subcutaneously with HEP3B cells tagged with a luciferase reporter protein. When the average tumor volume reached 80–120 mm^3^, mice were divided into two groups and received i.t. injections with either PBS or JURV at a dose of 1.0 × 10^7^ TCID_50_ (days 0, 7, and 14). (A) Tumor volume was recorded twice weekly until the humane endpoint, or end of the study (day 21). HEP3B tumors treated with PBS or JURV were harvested and analyzed for changes in protein expression. (B) Volcano plot of protein expression differences in HEP3B tumors treated with PBS vs. 1 × 10^7^ TCID_50_ of JURV. (C) 3D pie slices of the numbers of differentially expressed proteins (DEPs) in HEP3B tumors injected with PBS vs. 1 × 10^7^ TCID_50_ of JURV. (D) Heatmap of the top 20 DEPs upregulated or downregulated in HEP3B tumors injected with PBS vs. 1.0 × 10^7^ TCID_50_ of JURV. DEPs were determined using the limma-voom method as described in material and methods section. A fold-change |logFC| ≥ 1 and a false discovery rate (FDR) of 0.05 were used as a cutoff. The logFC was computed using the difference between the mean of log2(JURV) and the mean of log2(PBS), that is, mean of log2(JURV) – mean of log2(PBS). (E) Graph showing top-scoring canonical pathways significantly enriched by treatment with 1.0 × 10^7^ TCID_50_ of JURV in the HEP3B tumors.

Next, we evaluated whether the observed *in vitro* cell killing capacity of JURV (Figure 1) is associated with its capacity to induce an oncolysis-dependent tumor cell killing *in vivo*. We injected intratumorally (i.t.) three doses of JURV into human HEP3B xenografts. We used luciferase-tagged HEP3B cells to monitor tumor growth during the first 3 weeks of treatment. Bioluminescence imaging revealed significant tumor inhibition (*p* < 0.0001) in JURV-treated mice compared with phosphate-buffered saline (PBS) controls, evident from the first week post-injection (Figures S2A–S2C). We observed a significant (*p* < 0.0001) reduction in tumor growth (>90%) in the JURV-treated group (Figure 3A). However, while the HEP3B xenograft mice exhibited tumor reduction, we also noted some weight loss (Figure S2D), which could be due to tumor volume reduction. In addition, NOD scid mice, being severely immunocompromised, are susceptible to viral infections, which likely contributed to this effect as well. In contrast, in immunocompetent HCC models, JURV-treated mice maintained stable body weight, further supporting the safety and tolerability of JURV in hosts with intact immune systems as described elsewhere in this manuscript.

We have previously demonstrated that the responsiveness to type I IFN production (also shown in Figure S6) or viral kinetics *in vitro* by infected cancer cell lines does not always correlate with the *in vivo* efficacy of OVs.^13^ Consequently, we conducted a proteomics analysis of tumor tissues to identify changes, specifically focusing on proteins involved in the anti-viral pathway, following intratumoral delivery of JURV in HEP3B tumors. The analysis of 2,088 proteins showed that a storm of 160 differentially expressed proteins (DEPs) were upregulated, and 170 DEPs were downregulated in the JURV-treated vs. control group tumors (Figures 3B and 3C). Key upregulated proteins, including VIM,^15^ LCP1,^16^ COL6A3,^17^ HSPG2,^18^ NAMPT,^18^ and STAT1,^19^ are associated with the activation of the mTORC2/AKT pathway, whose inhibition reduces the expression of type I IFN genes (IFN-α/β) during TLR triggering (Figures 3D and 3E).

### Anti-tumor activity of JURV in a syngeneic HCC model

**Figure 4:**
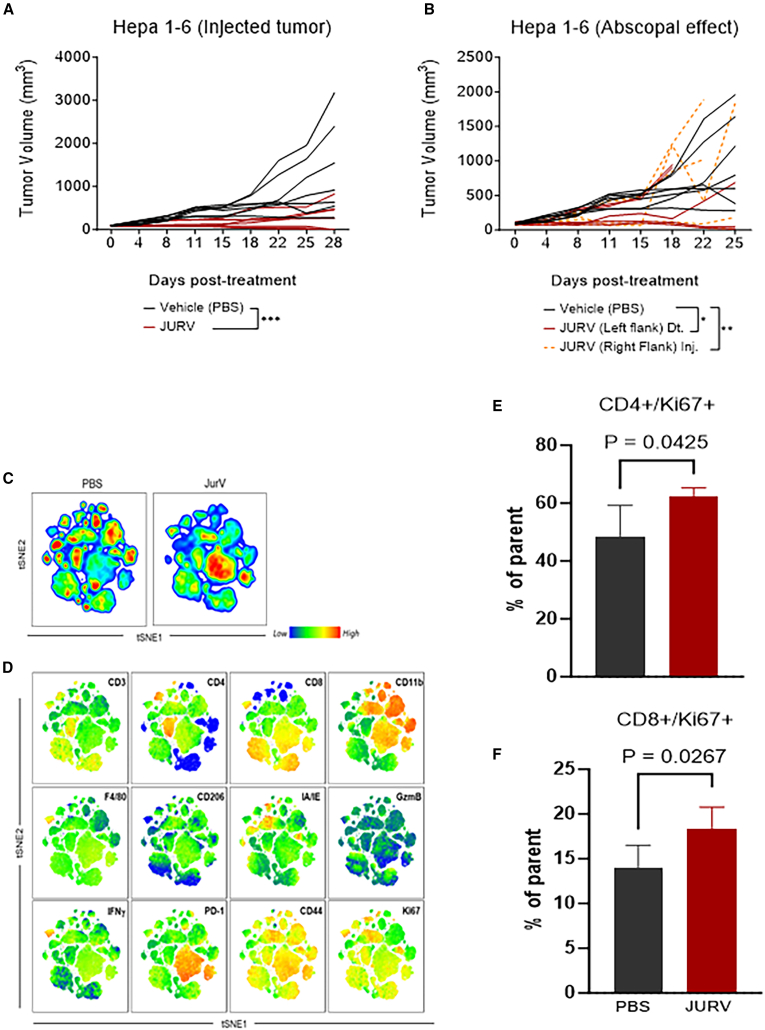
Evaluation of the anti-tumor efficacy of oncolytic JURV in an immuno-competent murine HCC model HEPA 1–6 cells were implanted into the right flanks of female C57BL6/J (strain no. 000664) (*n* = 7/group; Jackson Laboratory). (A) When the average tumor volume reached 80–120 mm^3^, mice were administered 50 μL i.t. injections containing PBS (vehicle) or 1 × 10^7^ TCID_50_ units of JURV were injected (inj.) into tumor-bearing mice at days 0, 7, and 14. Tumor volume was recorded twice weekly. Tumors were harvested at the end of the study for downstream analysis. (B) In the abscopal model (dual flanks), HEPA 1–6 cells (1 × 10^6^ cells/mouse) were first subcutaneously grafted into the right flanks and were categorized as “primary” tumors. Simultaneously, we performed distant HEPA 1–6 tumor grafts (1 × 10^6^ cells/mouse) into the left flanks of these mice. Mice in the dual-flank group received 50 μL i.t. injections of 1 × 10^7^ TCID_50_ units of JURV only on their right flanks once a week for 3 weeks. Data plotted as mean ± SD; ∗∗*p* < 0.001, ∗∗∗*p* < 0.0001. Area under the curve for tumor growth was compared by one-way ANOVA with Holm-Sidak correction for type I error. The first day of JURV or PBS injection was defined as day 0. t-SNE (t-distributed stochastic neighbor embedding) plot showing variable composition of tumor-infiltrating lymphocytes in JURV-treated tumors. Viable CD45 (12,500 events per tumor) were clustered by t-SNE. (C) Global cell density by t-SNE for each tumor treatment group. (D) Heatmap level of expression of each cellular marker across all groups. (E and F) Analysis of tumor-infiltrating immune cells following i.t. injection of oncolytic JURV in murine HCC tumors. The parent gate used is the live CD45+CD3+ population.

A subcutaneous syngeneic HEPA 1–6 HCC model was used to evaluate the anti-tumor efficacy of JURV. The treatment regimen included three i.t. doses of JURV within 3 weeks. A significant delay in tumor growth was observed in mice treated with JURV (*p* < 0.0001) compared with PBS-injected control (Figure 4A), with no adverse effects (Figure S3). In addition, to investigate further the potential abscopal effect and the broader systemic immune response triggered by JURV, we implanted bilateral Hepa 1–6 tumors subcutaneously on both flanks of the mice. JURV was administered i.t. exclusively to the right flank tumors. Interestingly, this treatment led to tumor regression on both the treated and untreated sides, indicating a potential systemic anti-tumor response (Figure 4B). However, we recognize the complexity of accurately evaluating the abscopal effect. Further studies are required to thoroughly assess JURV’s ability to induce local and systemic immune responses capable of eradicating distant tumors.

### Immune modulation by JURV treatment in the HCC model

We analyzed the changes in the immune landscape in murine HCC treated with JURV by flow cytometry. With t-SNE analysis (Figures 4C and 4D), we observed that JURV treatment-induced tumor growth delay (Figure 4A) was associated with a significantly altered TME to favor a more robust immune response. This effect was evidenced by increased markers of activated and proliferating T cells (CD44, Ki67), cytotoxic markers (CD8, GzmB), and IFN-γ production (Figures 4C–4F and S5A–S5J), with PD-1 expression suggesting a potentially active immune response. Our results indicate that JURV effectively recruits cytotoxic T lymphocytes and modulates immunosuppression, a key feature of durable immunotherapy responses.

### Multi-omics analysis identified fundamental molecular mechanisms of the anti-tumor activity of JURV *in vivo*

**Figure 5:**
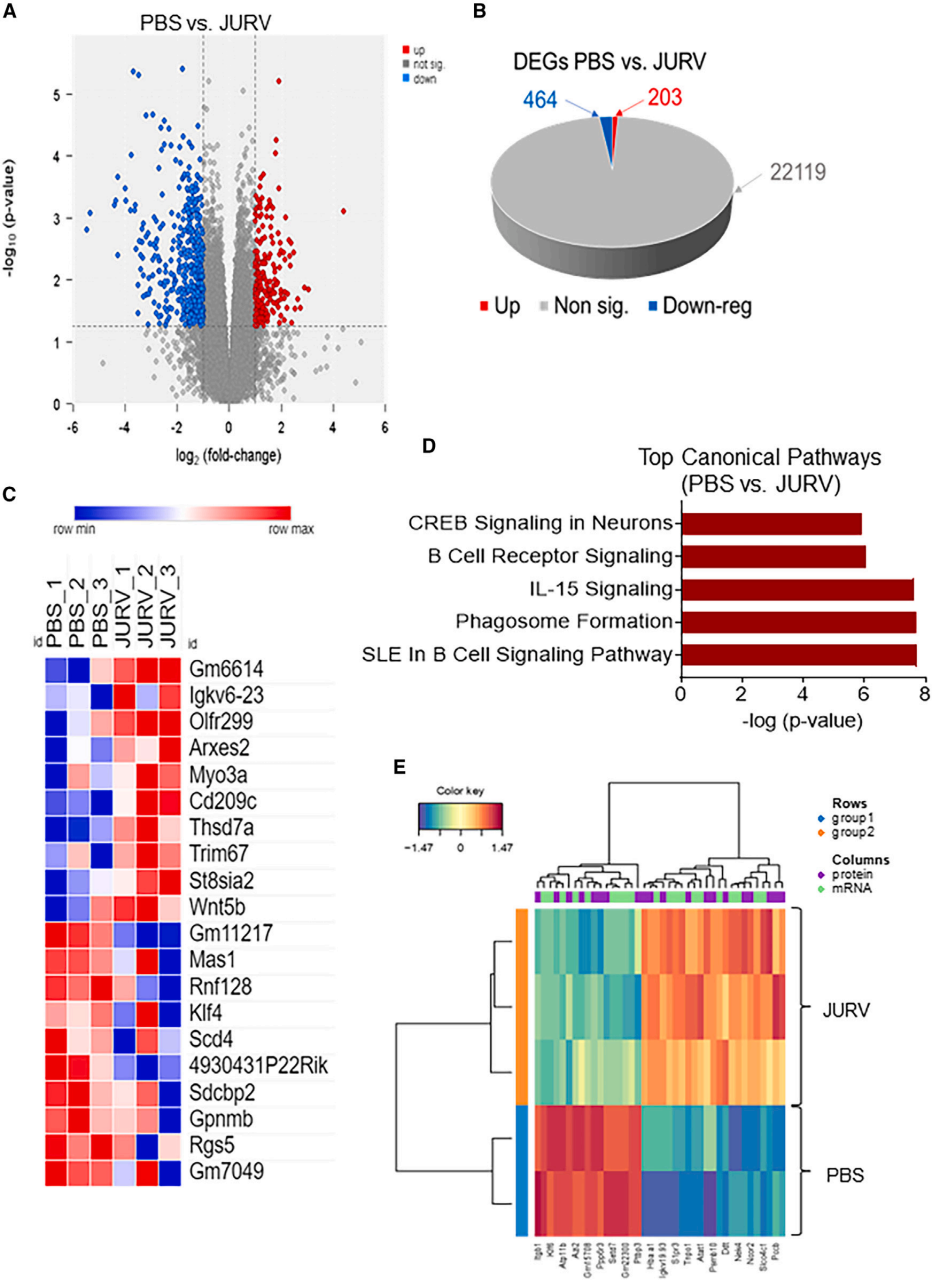
Proteogenomic changes in murine HCC injected with oncolytic JURV (A) Volcano plot of murine HCC tumor mRNA expression differences for PBS vs. JURV (1.0 × 10^7^ TCID_50_). (B) 3D pie slices of the numbers of differentially expressed genes (DEGs) between PBS vs. JURV. (C) Heatmap of the top 20 DEGs upregulated or downregulated in PBS vs. JURV. DEGs were determined using the limma-voom. (D) Graph showing top-scoring canonical pathways significantly enriched by treatment with PBS vs. JURV. A MixOmics supervised analysis was carried out between DEPs and DEGs based on Log2 fold change values. Log2 fold change of DEG × Log2 fold change of DEP > 0 with a *p* value of DEG and DEP < 0.05 were considered associated DEGs/DEPs. (E) DEG/DEP expression heatmap of the 30 most upregulated and downregulated features DEG/DEP in PBS vs. JURV.

Hepa 1–6 HCC tumors were subjected to transcriptional profiling to discern the gene and pathway alterations occurring following treatment with JURV, compared with controls treated with PBS. Differentially expressed genes (DEGs) were analyzed using the limma-voom method.^20^ Our data (Figures 5A and 5B) showed that, among the 22,786 genes, 203 DEGs were upregulated and 464 DEGs were downregulated (2-fold change >2, *p* < 0.055). Several of the top 10 upregulated DEGs, Myo3a,^21^ Cd209c,^22^ Trim67,^23^ St8sia2,^24^ and Wnt5b^25^ are associated with immune response pathways (Figure 5C). Many of the enriched cellular signaling pathways, such as the B cell receptor signaling, IL-15 signaling, and phagosome formation, identified by IPA analysis are related to the activation of the host’s innate and adaptive immune responses (Figure 5D). Furthermore, to better understand the mechanism of JURV-induced anti-tumor activity, we analyzed the DEPs and DEGs from the transcriptomic and proteomic data (Figure 5E). In the associated DEGs/DEPs, we identified the top 30 enriched features that are significantly upregulated or downregulated in the JURV group compared with the PBS-treated control group. Among the upregulated features, S1pr3,^26^ Tnpo1,^27^ Psmb1,^28^ Ddt,^29^ Ncor2,^30^ and Slc04c1^31^ have been identified in inflammation, host immune response against microorganisms (virus, bacteria), and tumorigenesis. These studies reveal potential molecular mechanisms involved in the JURV-induced anti-tumor activities.

### Synergistic effects of JURV and anti-PD-1 therapy in prolonging survival and inducing immune response in a metastatic orthotopic HCC mouse model

To comprehensively evaluate the therapeutic efficacy of oncolytic JURV across diverse TMEs, we employed distinct experimental approaches tailored to each model: i.t. injections for the non-metastatic Hepa 1–6 model and intraperitoneal (i.p.) injections for the metastatic RILWT model. Building on the observed effects of JURV in delaying tumor growth, modulating the TME, and activating immune effectors critical for anti-tumor immunity, we further investigated the synergistic potential of combining i.p. administration of JURV with anti-PD-1 therapy in an orthotopic RILWT mouse model.

**Figure 6:**
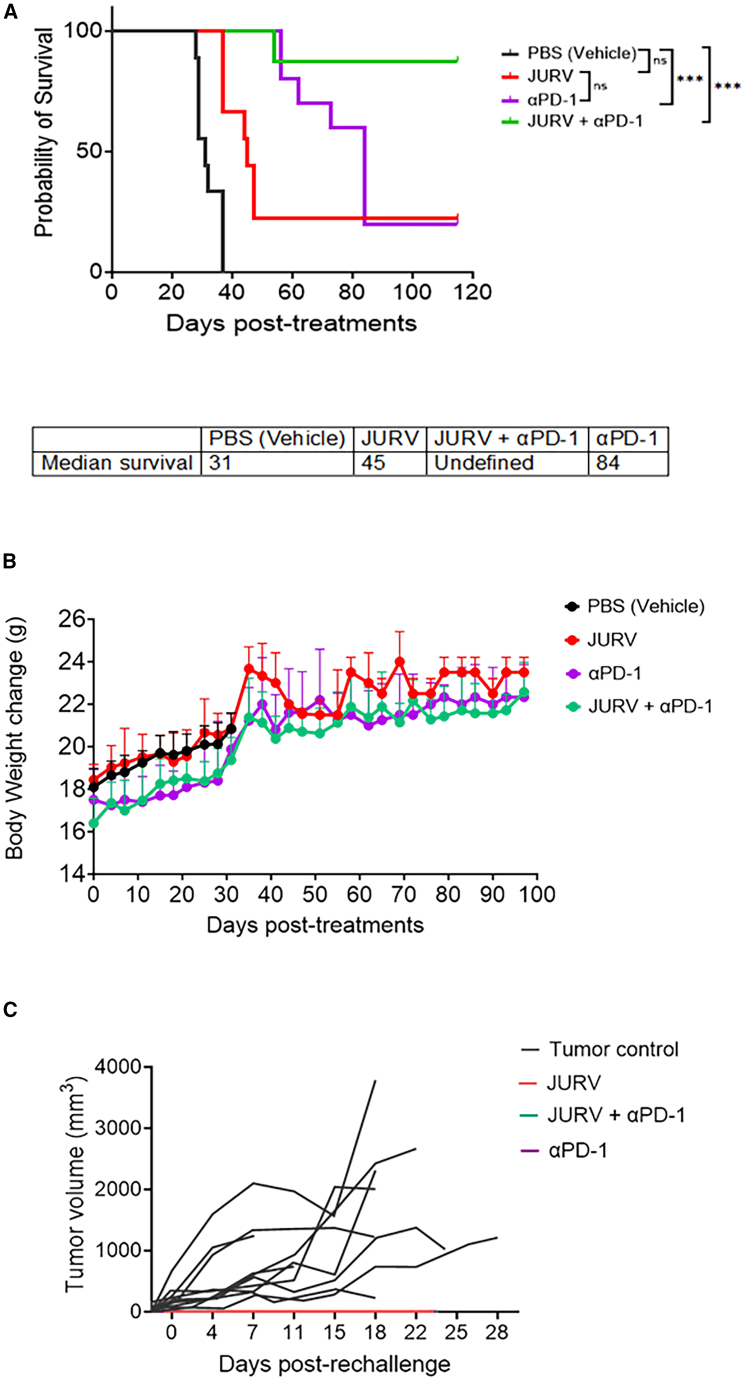
JURV synergizes with checkpoint inhibitors to significantly control tumor growth and prolong survival compared with single treatments in the metastatic HCC orthotopic mouse model (A) Kaplan-Meier survival curves illustrate the probability of survival over time for RILWT tumor-bearing mice (*n* = 10/group) treated with PBS (vehicle), JURV alone, anti-PD-1 antibodies alone, and the combination of JURV and anti-PD-1. Median survival times are indicated for each treatment group, with the combination therapy showing significantly extended survival compared with all other groups (*p* < 0.0001). (B) Body weight changes of the mice are plotted over time post-treatment, serving as an indirect measure of general health and treatment tolerability. Data points represent mean body weights with error bars indicating standard deviation. (C) Tumor growth post-rechallenge demonstrates individual tumor progression for each treatment cohort. JURV, αPD-1, and their combination notably inhibit tumor growth, which correlates with enhanced survival rates and suggests induction of tumor-specific immune responses. Statistical significance for survival rates was calculated using log rank (Mantel-Cox) tests, with the following notations: ns, not significant; ∗*p* < 0.05, ∗∗*p* < 0.01, ∗∗∗*p* < 0.001, ∗∗∗∗*p* < 0.0001. Tumor volume and body weight data were analyzed using repeated measures ANOVA with post hoc tests appropriate for multiple comparisons.

Employing immunocompetent C57BL6/J mice with RILWT HCC cells implanted orthotopically, we administered i.p. injections of JURV (1.0 × 10^7^ TCID_50_) weekly for 3 weeks from day 7 post-tumor implantation, either alone or combined with anti-PD-1 antibody (5 mg/kg given twice weekly for 3 weeks). Kaplan-Meier survival analysis showed significant improvements in survival for mice treated with anti-PD-1 antibodies (*p <* 0.0001), JURV (*p* = 0.0004), and notably the combination of JURV and anti-PD-1 antibodies (*p* < 0.0001), compared with PBS-treated controls (Figures 6A and 6B). The combination therapy notably outperformed both anti-PD-L1 antibody alone (*p* = 0.0151) and JURV alone (*p* = 0.0042) without inducing adverse clinical events (Figures 6A and 6B). Furthermore, RILWT-cured and treatment-naive mice were rechallenged subcutaneously with 5.0 × 10^5^ RILWT cells to evaluate long-term immunity. Interestingly, all mice previously treated with JURV, anti-PD-1, or their combination, successfully rejected the implanted RILWT cells, contrasting with the tumor development in treatment-naive mice (Figure 6C). These findings suggest the induction of a robust tumor-specific immune response by the treatments highlighting the potential of JURV in combination with anti-PD-1 therapy as a potent strategy for HCC treatment.

## Discussion

In this study, we have demonstrated the oncolytic efficacy of JURV in targeting murine and human HCC cell lines *in vitro*, as well as its capability to delay tumor growth and prolong survival in murine cancer models of HCC. Our data show that JURV modulated the TME by enhancing the infiltration of cytotoxic T cells and recruiting diverse immune effectors. When used in combination with anti-PD-1 antibodies, JURV greatly enhances tumor regression and improves survival rates in orthotopic HCC models. This survival benefit remained effective when surviving mice were rechallenged with subsequent tumor implantations, strongly indicating a tumor-specific immune response. This work further investigated the safety profile of JURV, underscoring its lack of neurotoxic and hepatotoxic effects, thus making it a promising candidate for oncolytic viral therapy. This safety, combined with its effectiveness, was demonstrated in HEP3B xenografts, where JURV’s anti-tumor activity led to the complete eradication of human HCC in tumor-bearing mice. This outcome correlated with our *in vitro* cytotoxicity assays and the activation of IFN-associated proteins, as described in our earlier publication.^13^^,^^32^ Moreover, our data reveal the protective effect of exogenous type I IFN, which reduces JURV-induced cell killing in a dose-dependent manner. This suggests that normal tissues with intact IFN responses are likely protected from viral infection, further supporting JURV’s tumor specificity and safety.

Our comprehensive analysis of proteomic and transcriptomic data uncovered various molecular pathway alterations and changes in gene expression in HEPA 1–6 tumors following treatment with i.t. injections of JURV. Transcriptional profiling identified several DEGs associated with immune response pathways, such as Myo3a, Cd209c, Trim67, St8sia2, and Wnt5b. Enrichment analysis highlighted major immune-related signaling pathways, including B cell receptor and IL-15 signaling. By integrating transcriptomic and proteomic data, we observed the upregulation of proteins such as S1pr3, Tnpo1, and Psmb10, which are involved in inflammation, immune response, and tumorigenesis.

However, we acknowledge several limitations in the models used. The subcutaneous Hepa 1–6 tumor model, while providing important insights into localized tumor-immune interactions, does not fully replicate the complex TME or metastatic behavior typical of HCC. In addition, the use of human cell lines in xenograft models presents challenges due to species-specific immune system differences, which may affect the translational relevance of our findings. To address these limitations, future studies employing orthotopic or patient-derived models are necessary to validate our observations and refine therapeutic strategies.

Our results also align with the concept of locoregional oncolytic virotherapy as reported in other therapies.^33^ It shows that JURV-mediated oncolysis effectively induces tumor growth delays in both primary and distant tumors, demonstrating its ability to trigger an abscopal effect that is less commonly observed in other therapies for HCC.^34^

In summary, we demonstrated that JURV effectively induces cancer cell death and stimulates anti-tumor immunity in HCC. Moreover, we showed that the combination of JURV with anti-PD-1 antibodies provides additional survival benefits in preclinical HCC models. This study not only highlights the potential of JURV as a potent therapeutic option for HCC treatment but also introduces an innovative strategy with the potential to overcome challenges such as low immunogenicity and immunosuppression safely and potently. The addition of JURV in the field of oncolytic viral therapy promises to broaden the clinical application of OVs in cancer treatment, providing new avenues for therapy optimization.

## Materials and methods

### Virus

The procedure used for JURV recovery was as in Lawson et al. ^35^ In short, 6-well plates were used to plate BHK cells at a density of 5 × 10^5^ cells/well. At an MOI of 10, the cells were infected with a vaccinia virus that encodes T7 polymerase. Following 1 h incubation, excess vaccinia was removed and cells were transfected with 2 μg pJURV, 1 μg pN, 0.8 μg pP, and 0.4 μg pL (the N, P, and L plasmids were constructed in the pCI vector) using 12.5 μL of Lipofectamine LTX transfection reagent (Life Technologies, Grand Island, NY) following the manufacturer’s instructions. The cells were incubated in Opti-MEM Reduced-Serum Medium (Gibco) at 37°C for 48 h. The cells were cultured in Opti-MEM Reduced-Serum Medium (Gibco) for 48 h at 37°C. The culture medium was taken out after 48 h, twice filtered through a 0.2-μm filter, and then placed on top of fresh BHK cells in a 6-well plate. After 48 h, the culture medium was taken out, centrifuged at a low speed, filtered through a 0.2-μm filter, titrated on new Vero cells, and kept in storage at −80°C.

### Cell lines

This study used a panel of three human HCC cell lines: HEP3B (ATCC, cat. no. HB-8064), PLC, HuH7 (RRID: CVCL_0336), and two murine HCC cell lines: HEPA 1–6 (ATCC, cat. no. CRL-1830, RRID: CVCL_0327) and R1LWT (RRID: CVCL_B7TK). We also used several murine solid tumor cells, including colon carcinoma cells (CT26, ATCC, cat. no. CRL-2638, RRID: CVCL_7256), skin melanoma cells (B16-F10, ATCC, cat. no. CRL-6475, RRID: CVCL_0159), and prostate cancer cells (RM-1, ATCC, cat. no. CRL-3310, RRID: CVCL_B459). All cell lines were cultured at 37°C with 5% CO_2_ in medium supplemented with antibiotic agents (100 μg/mL penicillin and 100 μg/mL streptomycin). HEP3B, PLC, and HuH7 were maintained in Dulbecco’s modified Eagle’s medium (DMEM) with 10% fetal bovine serum (FBS). We maintained HEPA 1–6, RILWT, BHK-21 (ATCC, cat. no. CCL-10, RRID: CVCL_1915), and Vero cells (ATCC, cat. no. CCL-81, RRID: CVCL_0059) in DMEM with 10% FBS. BHK-21, Vero, HEP3B, PLC, HuH7, HEPA 1–6, CT26, and BF16-F10 cells were obtained from the American Type Culture Collection (Manassas, VA). The RILWT cell line derived from RIL-175 cells was from Dan G. Duda, PhD, Massachusetts General Hospital, Boston, MA.

### Amplification of JURV

Viral amplification was done by infecting confluent (∼80%) Vero cells in T-175 flasks of JURV at an MOI of 0.001. At 48 h post-infection or when cytopathic effects were observable, supernatants of virus-infected cells were collected from the flasks. The viral stocks were purified using 10%–40% sucrose-density gradient ultracentrifugation followed by dialysis. The titer (TCID_50_) of the rescued virus was determined by the Spearman-Kärber algorithm using serial viral dilutions in BHK-21 cells. BHK-21 (ATCC, cat. no. CCL-10, RRID: CVCL_1915) and Vero cells (ATCC, cat. no. CCL-81, RRID: CVCL_0059) were obtained from the American Type Culture Collection.

### Cell viability assays

For all cytotoxicity assays (96-well format), 1.5 × 10^4^ HEP3B, PLC, HuH7, HEPA 1–6, or RILWT cells were infected with JURV at the indicated MOIs of 10, 1, or 0.1 in serum-free Gibco Minimum Essential Medium (Opti-MEM). Cell viability was determined using a Cell Titer 96 AQueous One Solution Cell Proliferation Assay (Promega, Madison, WI). Data were generated from six replicates from two independent experiments ± SEM.

### Crystal violet assays

Five hundred thousand HEP3B, PLC, HuH7, HEPA 1–6, or RILWT cells were infected with oncolytic JURV in 6-well plates at an MOI of 0.1 for 1 h. Supernatants of virus-infected cells were removed, and cells were washed with PBS and incubated at 37°C until analysis. At 72 h after infection, cells were fixed with 5% glutaraldehyde and stained with 0.1% crystal violet to visualize the cellular morphology and remaining adherence indicative of cell viability. Pictures of representative areas were taken.

### One-step viral growth kinetics

Two hundred thousand HCC cells were plated in each well of a 6-well plate in 2 mL of complete DMEM. After allowing cells to rest overnight, we infected them with JURV at an MOI of 0.1 for 1 h. Supernatants of virus-infected cells were removed, cells were washed with PBS, and fresh medium was added. At 10, 24, 48, and 72 h, the supernatant was collected and stored at −80°C. Viral titers (PFU/mL) were determined with serial dilutions of the supernatant on Vero cells. Data were generated as means of two independent experiments ± SEM.

### Flow cytometry antibody analysis

The following antibodies were used for flow cytometry analysis: CD45-FITC (BD Biosciences, cat. no. 553079), CD3-BUV395 (BD Biosciences, cat. no. 563565, RRID: AB_2738278), CD4-BUV737 (BD Biosciences, cat. no. 612761, RRID: AB_2870092), CD8-Percp-Cy5.5 (Themo Fisher Scientific, cat. no. 45-0081-82, RRID: AB_1107004), CD44-BV711 (BioLegend, cat. no. 103057, RRID: AB_2564214), CD335-PE/Dazzle594 (BioLegend, cat. no. 137629 [also 137630], RRID: AB_2616665), PD-1-PE (BD Biosciences, cat. no. 551892, RRID: AB_394284), Ki67∗-BV605 (BioLegend, cat. no. 652413, RRID: AB_2562664), Granzyme B∗-APC (BioLegend, cat. no. 372204 [also 372203], RRID: AB_2687028), IFN-γ∗-BV421 (BD Biosciences, cat. no. 563376, RRID: AB_2738165), CD11b-PE-Cy7 (BioLegend, cat. no. 101216 [also 101215], RRID: AB_312799), F4/80-BV51.00 (BioLegend, cat. no. 123135, RRID: AB_2562622), CD206-AF700 (BioLegend, cat. no. 141734 [also 141733], RRID: AB_2629637), I-A/I-E-BV786 (BD Biosciences, cat. no. 743875, RRID: AB_2741826), and L/D-efluor780 (cat. no. 65-0865-18, eBioscience).

### Animal studies

Female mice C57BL/6J (RRID: IMSR_JAX: 000664), BALB/cJ (RRID: IMSR_JAX:000651), and NOD.Cg-Prkdc^scid^/J (RRID: IMSR_JAX:001303) were purchased from Jackson Laboratories at age 6–8 weeks. Male C57BL6/J mice (RRID: IMSR_JAX:000664) were also obtained from Jackson Laboratories. All mice were housed at the Division of Laboratory Animal Medicine at the University of Arkansas for Medical Sciences (UAMS), which employs a full staff of veterinarians and veterinary technicians who supervised and assisted in animal care throughout the studies. All animal studies were approved by the Institutional Animal Care and Use Committee at UAMS.

### Analysis of virus-induced adverse events in mice

Female C57BL/6J mice (RRID: IMSR_JAX:000664) (*n* = 6 mice/group) were administered PBS, a moderately high viral dose (1.0 × 10^7^ TCID_50_), or a high viral dose (1.0 × 10^8^ TCID_50_) i.n. (25 μL in each nostril) or i.v. (50 μL/mouse). Body weight, temperature, behavior, and clinical signs were monitored by a board-certified veterinarian at least three times a week to detect any signs of toxicity. At 3 days post-infection, three mice per group were sacrificed, and blood and animal tissues (brain, liver, and spleen) were collected and subjected to hematoxylin and eosin staining to assess short-term toxicity and viral biodistribution. The remaining mice were monitored for 30 days.

### HEP3B xenograft model

Female NOD.Cg-*Prkdc*^*scid*^/J mice (RRID: IMSR_JAX:001303) were subcutaneously inoculated with HEP3B cells (ATCC, cat. no. HB-8064) expressing a firefly luciferase reporter gene on the right flanks (*n* = 6–7/group). When the average tumor volume reached 80–120 mm^3^, mice were administered 50 μL i.t. injections of JURV (1.0 × 10^7^ TCID_50_) or 50 μL of PBS (controls) once weekly for 3 weeks. Tumor volume was measured twice weekly until the end of the study (day 21), or the humane endpoint as described above. We also recorded mouse body weight and clinical observations twice per week.

### Bioluminescence imaging

Tumor-bearing (HEP3B) mice were anesthetized with isoflurane and imaged once a week (days 0, 7, and 14) with an IVIS Xenogen imaging system to assess virus-induced changes in tumor growth. Anesthesia was induced in an induction chamber (2%–5% isoflurane), after which the mice were placed in the imaging instrument and fitted with a nose cone connected to a vaporizer to maintain the isoflurane concentration (0.5%–2%) during the procedure. This range of concentrations produces a level of anesthesia that prevents animal movement during scanning. If the respiratory rate accelerates or slows, the isoflurane concentration is increased or decreased. We used a heated animal bed, heating pads, and, if necessary, a heating lamp to ensure that body temperature was maintained both before imaging and during the procedure. Each mouse received an i.p. injection of D-luciferin (Sigma-Aldrich, no. L9504; 50 mg/kg body weight in the volume of 5 μL/g of body weight, prepared in sterile water). Anesthetized mice were placed into the IVIS Xenogen imaging system on their stomachs. Imaging of each group of mice took less than 10 min. This was a non-invasive imaging procedure, and no restraints were needed.

### *In vivo* efficacy of the oncolytic JURV in a syngeneic SQ mouse model of HCC

To evaluate the *in vivo* therapeutic efficacy of oncolytic JURV in a syngeneic mouse HCC model, we injected 1 × 10^6^ HEPA 1–6 cells in 100 μL of cold RPMI into the right flanks of immunocompetent female C57BL6/J mice (*n* = 7–8/group; Jackson Laboratory) using 1 mL syringes. Mice were monitored weekly for palpable tumors or any changes in appearance or behavior. When average tumors reached a treatable size (80–120 mm^3^), mice were randomized into the respective study groups—PBS (controls) and JURV. Dosing began within 24 h of randomization. Depending on the treatment regimen, mice were administered 50 μL i.t. injections of either PBS or JURV (1 × 10^7^ TCID_50_ units) on days 0, 7, and 14. To establish syngeneic bilateral HCC tumors (dual flanks), in additional groups of mice, HEPA 1–6 cells (1 × 10^6^ cells/mouse) were first subcutaneously grafted into the right flanks (resulting in tumors at ∼14 days) and categorized as “primary” tumors. Simultaneously, we performed distant HEPA 1–6 tumor graft injections (1 × 10^6^ cells/mouse) into the left flanks of these mice. Mice in the dual-flank groups only received 50 μL i.t. injections of 1 × 10^7^ TCID_50_ units of JURV on their right flanks once a week for 3 weeks. Tumor volume and body weight were measured twice weekly using a digital caliper and balance following randomization and initiation of treatment. Tumor volume was calculated as (longest diameter × shortest diameter^2^)/2. During the first week of treatment and after each injection, mice were monitored daily for signs of recovery for up to 72 h. Mice were euthanized when body weight loss exceeded 20%, when tumor size was larger than 2,000 m³, or for adverse effects of treatment. Mice were sacrificed 28 days following the first JURV dose administration, at which time tumors and blood were collected for downstream analysis.

### *In vivo* efficacy of the oncolytic JURV in a syngeneic orthotopic mouse model of HCC

To evaluate the *in vivo* therapeutic efficacy of oncolytic JURV in a syngeneic mouse orthotopic HCC model, 1.0 × 10^6^ luciferase-expressing RILWT cells were surgically implanted into one of the lobes of the liver of a syngeneic orthotopic HCC mouse model. Following 14 days after tumor implantation, mice were randomized (*n* = 10/group) and grouped. To determine the safety and efficacy of the JURV (1.0 × 10^7^ TCID_50_) and/or anti-mPD-1 (5 mg/kg, Bio X Cell, cat. no. BP0273, RRID: AB_2687796) were administered i.p. Tumor size was measured by bioluminescent imaging 14 days after tumor implantation for animal randomization and once weekly for 60–90 days. Body weight was measured twice weekly. During the first week of treatment and after each injection, mice were monitored daily for signs of recovery for up to 72 h. Mice were euthanized when body weight loss exceeded 20% or for adverse effects of treatment. Mortality during the survival study was assessed using the log rank test to compare the differences in Kaplan-Meier survival curves. RILWT-cured or treatment-naive C57BL/6J mice were rechallenged by subcutaneously inoculating 5.0 × 10^5^ RILWT cells. Tumor growth was monitored for 30 days post-implantation.

### Analysis of tumor-infiltrating immune cells

Hepa 1–6 tumors (*n* = 3 samples/group) were excised and dissociated on day 18, 3 days after the last JURV injection, using a mouse tumor dissociation kit (Miltenyi, cat. no. 130-096-730) with a gentleMACS Octo Dissociator (Miltenyi) according to the manufacturer’s protocol. CD45^+^ cells were isolated with mouse CD45 (TIL) microbeads (Miltenyi). Cells were incubated with Fixable Viability Stain 510 for 15 min at 4°C, followed by anti-Fc blocking reagent (BioLegend, cat. no. 101320) for 10 min before surface staining. Cells were stained, followed by data acquisition with a BD LSRFortessa X-20 flow cytometer. All antibodies (Table S1) were used following the manufacturer’s recommendation. Fluorescence Minus One control was used for each independent experiment to establish gating. For intracellular staining of granzyme B, cells were stained using an intracellular staining kit (Miltenyi), and analysis was performed using FlowJo (TreeStar). Forward scatter and side scatter cytometry were used to exclude cell debris and doublets.

### RNA sequencing of murine HCC tumors

Hepa 1–6 (*n* = 3 samples/group) FFPE scrolls were processed for DNA and RNA extraction using a Quick-DNA/RNA FFPE Miniprep Kit with on-column DNase digestion for the RNA preps (cat. no. R1009, Zymo Research). RNA was assessed for mass concentration using the Qubit RNA Broad Range Assay Kit (cat. no. Q10211, Invitrogen) with a Qubit 4 fluorometer (cat. no. Q33238, Invitrogen). RNA quality was assessed with a Standard Sensitivity RNA Analysis Kit (cat. no. DNF-471-0500, Agilent) on a Fragment Analyzer System (cat. no. M5310AA, Agilent). Sequencing libraries were prepared using TruSeq Stranded Total RNA Library Prep Gold (cat. no. 20020599, Illumina). RNA DV200 scores were used to determine fragmentation times. Libraries were assessed for mass concentration using a Qubit 1X dsDNA HS Assay Kit (cat. no. Q33231, Invitrogen) with a Qubit 4 fluorometer (cat. no. Q33238, Invitrogen). Library fragment size was assessed with a High Sensitivity NGS Fragment Analysis Kit (cat. no. DNF-474-0500, Agilent) on a Fragment Analyzer System (cat. no. M5310AA, Agilent). Libraries were functionally validated with a KAPA Universal Library Quantification Kit (cat. no. 07960140001, Roche). Sequencing was performed to generate paired-end reads (2 × 100 bp) with a 200-cycle S1 flow cell on a NovaSeq 6000 sequencing system (Illumina).

### Bioinformatics analysis

We examined the mRNA and protein expression profiles of Hepa 1–6 tumors treated with PBS, JURV, anti-PD-1, or JURV + anti-PD-1. Three replicates were used to analyze each of the untreated (PBS) and treated groups. The tumor samples were sequenced on an NGS platform. The files containing the sequencing reads (FASTQ) were then tested for quality control using MultiQC.^36^ The Cutadapt tool trims the Illumina adapter and low-quality bases at the end. After the quality control, the reads were aligned to a mouse reference genome (mm10/GRCm38) with the HISAT2 aligner,^37^ followed by counting reads mapped to RefSeq genes with feature counts. We generated the count matrix from the sequence reads using HTSeq-count.^38^ Genes with low counts across the samples affect the false discovery rate, thus reducing the power to detect DEGs; thus, before identifying DEGs, we filtered out genes with low expression utilizing a module in the limma-voom tool.^39^ Then, we normalized the counts by using TMM normalization,^40^ a weighted trimmed mean of the log expression proportions used to scale the counts of the samples. Finally, we fitted a linear model in limma to determine DEGs and expressed data as mean ± standard error of the mean. All *p* values were corrected for multiple comparisons using Benjamini-Hochberg FDR adjustment. After identifying DEGs, enriched pathways were performed using the Ingenuity Pathway Analyses (IPA) tool to gain biological insights. The statistical difference between groups was assessed using the nonparametric Mann-Whitney U test R module.

### Integration of transcriptomics and proteomics

The limma-normalized transcript expression levels and the normalized protein intensities were integrated using two independent methods. Firstly, the mixOmics package (Omics Data Integration Project R package, version 6.1.1) was implemented to generate heatmaps of the associated DEPs/DEGs as described previously.^41^ Secondly, the MOGSA package was used to generate heatmaps of the top 30 upregulated or downregulated DEPs/DEGs between the various groups.^42^

### Statistical analysis

All numerical variables were summarized using mean ± standard error. A one-way ANOVA model assessed the association of the numerical variable to an experiment factor. Post hoc means were compared between experiment groups after adjusting for multiple comparisons using Turkey’s method. Sequencing data were analyzed after controlling for false discovery rate using a Benjamini-Hochberg method. Time-to-event data were analyzed using Kaplan-Meier curves and compared between groups using a log rank test. Paired comparisons were conducted using paired t tests and/or Wilcoxon signed rank tests. Statistical analyses were performed using GraphPad Prism (RRID: SCR_002798). *p* values <0.05 were considered statistically significant.

## Data and code availability

The RNA sequencing data are freely available via GEOGSE199131, and the proteomics data are available via ProteomeXchange with the identifier PXD035806.

## Acknowledgments

We thank the personnel of the DNA Damage and Toxicology, Proteomics, Genomics, and Bioinformatic Cores at the University of Arkansas for Medical Sciences for their assistance during these studies. We also thank Dr. Musa Gabere for his assistance in analyzing the proteomic data. This work was supported by the 10.13039/100000002National Institutes of Health (NIH) through a 10.13039/100000054National Cancer Institute (NCI) grant (CA234324 to B.M.N.), an NIH New Innovator Award (DP2CA301099 to B.M.N.), a grant from the 10.13039/100000043American Association for Cancer Research (AACR) to B.M.N., the Winthrop P. Rockefeller Cancer Institute and the Barton Pilot Award program of UAMS College of Medicine to B.M.N. The UAMS Bioinformatics Core Facility is supported by the Winthrop P. Rockefeller Cancer Institute and NIH/10.13039/100000057NIGMS grant P20GM121293. This research is supported in part by a seed grant from the Vice Chancellor of Research & Innovation at UAMS. The IDeA National Resource for Quantitative Proteomics is supported by 10.13039/100000057NIGMS grant R24GM137786. Its contents are solely the responsibility of the authors and do not necessarily represent the official views of the NIH.

## Author contributions

M.Z.T., A.B., M.J.B., M.J.C., and B.M.N. contributed to the study concept and design, data acquisition, data analysis, data interpretation, and manuscript drafting. M.Z.T., Y.Z., M.T., C.S.S., J.C.C., C.D., O.B., A.L.G., R.S.S., M.E.F.-Z., C.Y.C., D.G.D., B.M., O.M., N.M.E., S.R.P., J.Y., and T.J.K. contributed to data acquisition, data analysis, data interpretation, drafting, and critical revision of the manuscript. C.L.W., D.A., A.G., and S.D.B. contributed to bioinformatic analysis. All authors approved the final, submitted version of the manuscript.

## Declaration of interests

The authors declare no competing interests.

## Supplemental information

Document S1. Figures S1–S6 and Table S1

Document S2. Article plus supplemental information
