# Supplementary material for: Enhancing immune response and survival in hepatocellular carcinoma with novel oncolytic Jurona virus and immune checkpoint blockade
Source: Mol Ther Oncol. 2024 Nov 26;32(4):200913. doi: 10.1016/j.omton.2024.200913 (PMC11697550; doi:10.1016/j.omton.2024.200913)
Supplement: Supplementary file 1 — Document S1. Figures S1–S6 and Table S1 [file mmc1.pdf]

OMTON, Volume *32*

Supplemental information

Enhancing immune response and survival in hepatocellular carcinoma with novel oncolytic Jurona virus and immune checkpoint blockade

Mulu Z. Tesfay, Yuguo Zhang, Khandoker U. Ferdous, Mika A. Taylor, Aleksandra Cios, Randal S. Shelton, Camila C. Simoes, Chelsae R. Watters, Oumar Barro, Natalie M. Elliott, Bahaa Mustafa, Jean Christopher Chamcheu, Alicia L. Graham, Charity L. Washam, Duah Alkam, Allen Gies, Stephanie D. Byrum, Emmanouil Giorgakis, Steven R. Post, Thomas Kelly, Jun Ying, Omeed Moaven, Chiswili Y. Chabu, Martin E. Fernandez-Zapico, Dan G. Duda, Lewis R. Roberts, Rang Govindarajan, Mitesh J. Borad, Martin J. Cannon, Alexei G. Basnakian, and Bolni M. Nagalo

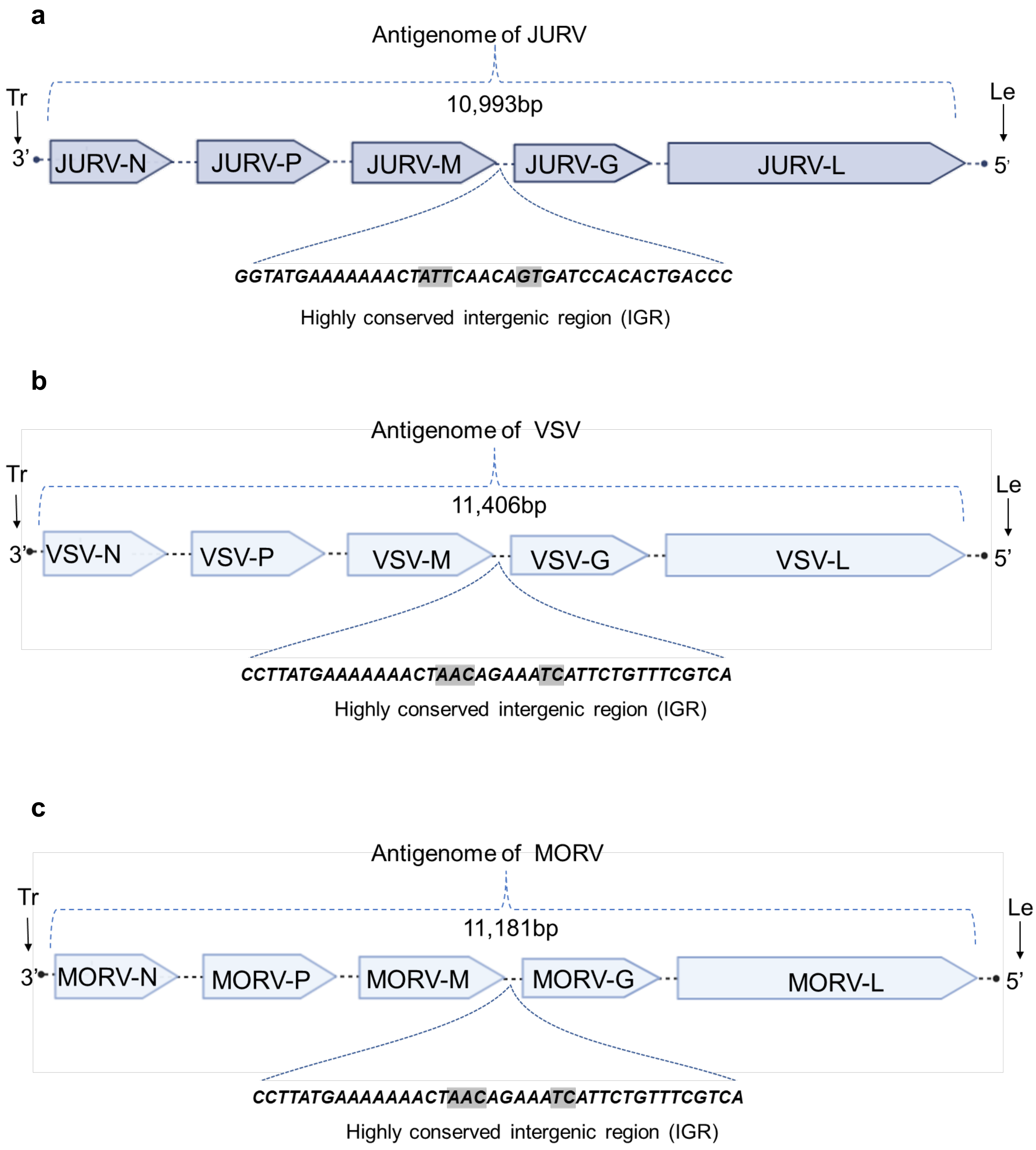

Figure S1. Schematic representation of the antigenomic structure of JURV, VSV, and MORV
The antigenome of JURV is typical of that of Rhabdovirus and consists of 5 major genes from the 3' to 5' antigenomic direction: nucleoprotein (JURV-N), phosphoprotein (JURV-P), matrix (JURV-M), glycoprotein (JURV-G), polymerase (JURV-L). Schematic representation of the 3' to 5' antigenomic organization of lab adapted a) Jurona virus (JURV), b) Vesicular stomatitis virus (VSV), and c) Morreton virus (MORV). The gene junctions' intergenic region (IGR) differed between the 3 viruses.

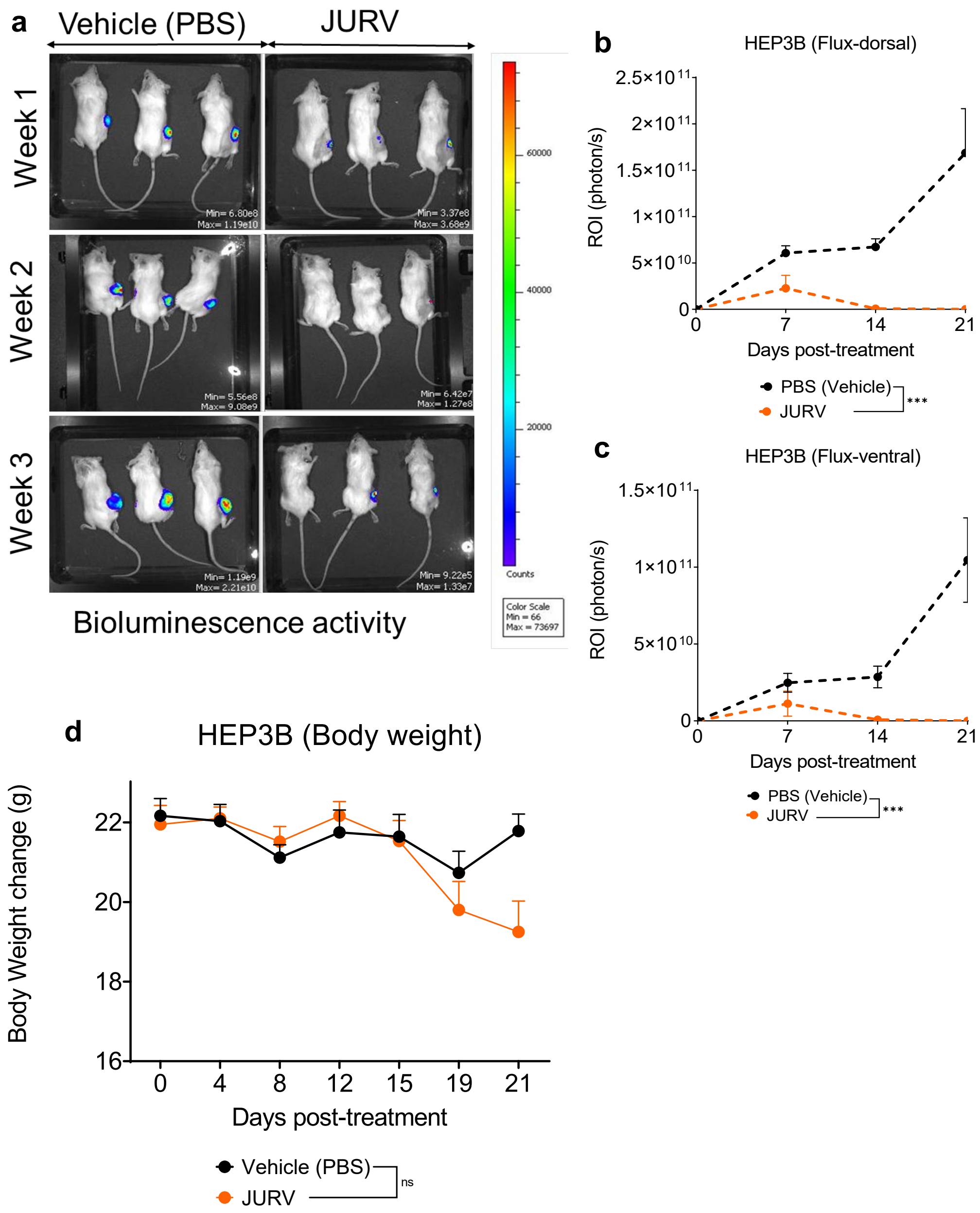

Figure S2. Oncolytic JURV induces a robust virus-mediated oncolysis dependent tumor growth delay in JURV in Hep3B Xenografts. Female NOD.Cg-Prkdcscid/J (Strain #:001303) mice (n=6-7/group) were inoculated subcutaneously with human luciferase-expressing HEP3B cells. (a) Photon emission from mice with subcutaneous HEP3B tumors. When the average tumor volume reached 80-120 mm3, mice were divided into two groups and received IT injections with either PBS or JURV (1.0 x 107 TCID50) on days 0, 7, and 14. Photon counts were obtained on weeks 1, 2, and 3. Photon emission at the right dorsal flanks (b) and ventral (c) were significantly (P<0.005) reduced among JURV-treated mice compared to PBS controls.

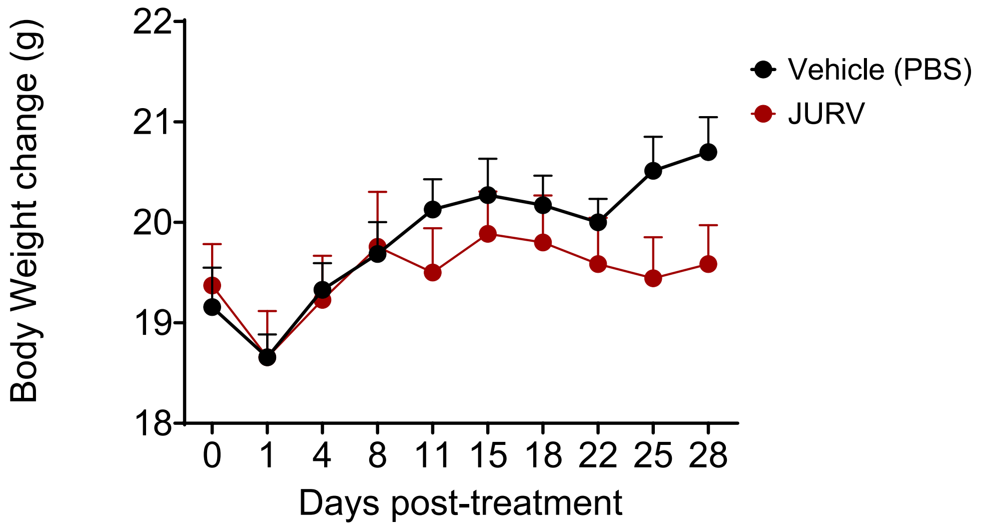

Figure S3. Changes in body weight of Hepa 1-6 tumor-bearing mice Treated with oncolytic JURV

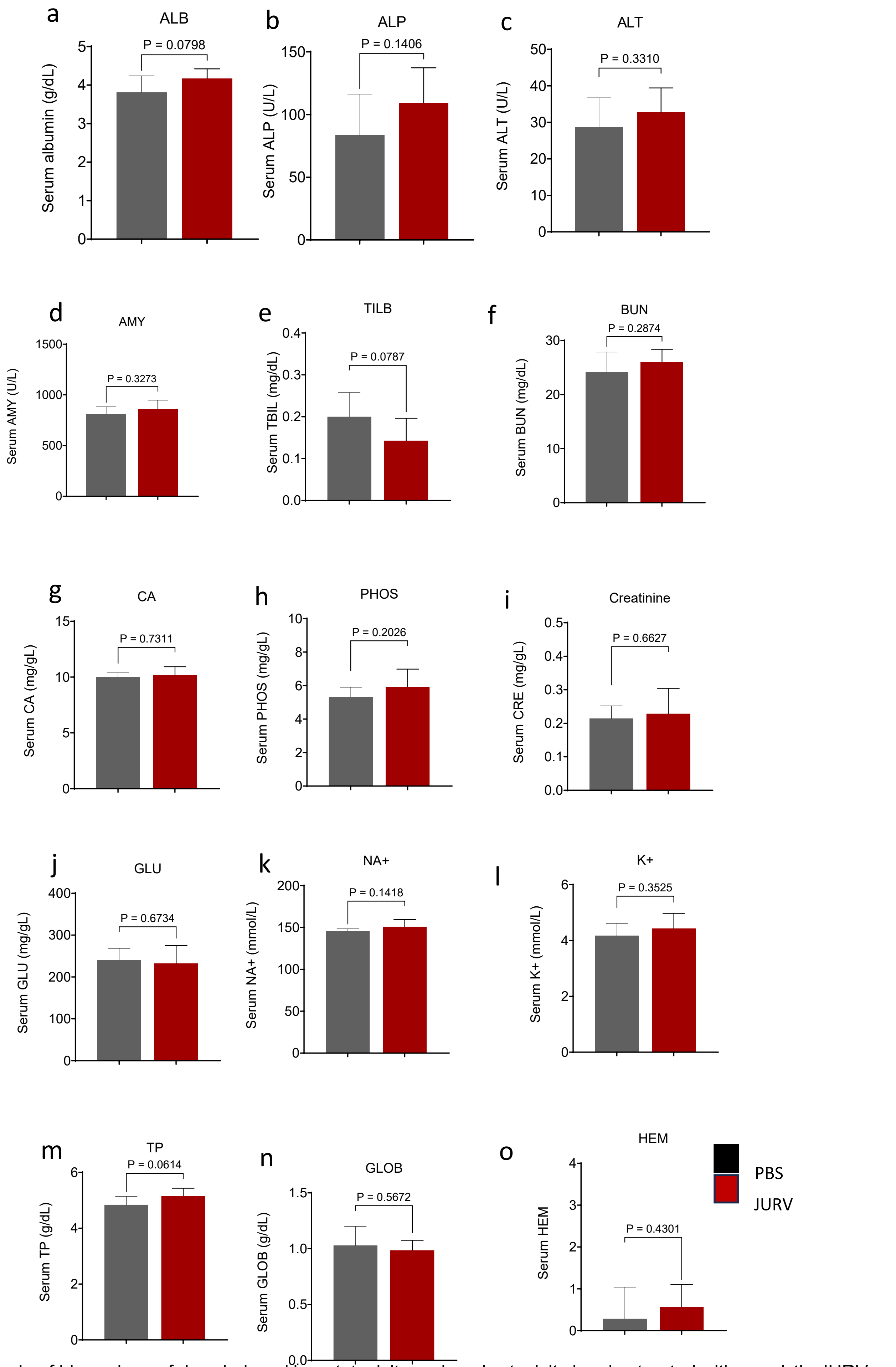

Figure S4. Analysis of biomarkers of drug-induced hepatotoxicity and nephrotoxicity in mice treated with oncolytic JURV. Changes in the serum biomarkers of drug-induced toxicity, including albumin (a, ALB), alkaline phosphatase (b, ALP), alanine aminotransferase (c, ALT), amylase (d, AMY), total bilirubin (e,TILB), blood urea nitrogen (f, BUN), calcium (g, CA), phosphate (h, PHOS), creatinine (i), glutamine (j, GLU), sodium (k, NA+), potassium (l, K+), total protein (m, TP), serum globulin (n, GLOB), and serum hemoglobulin (o, HEM).

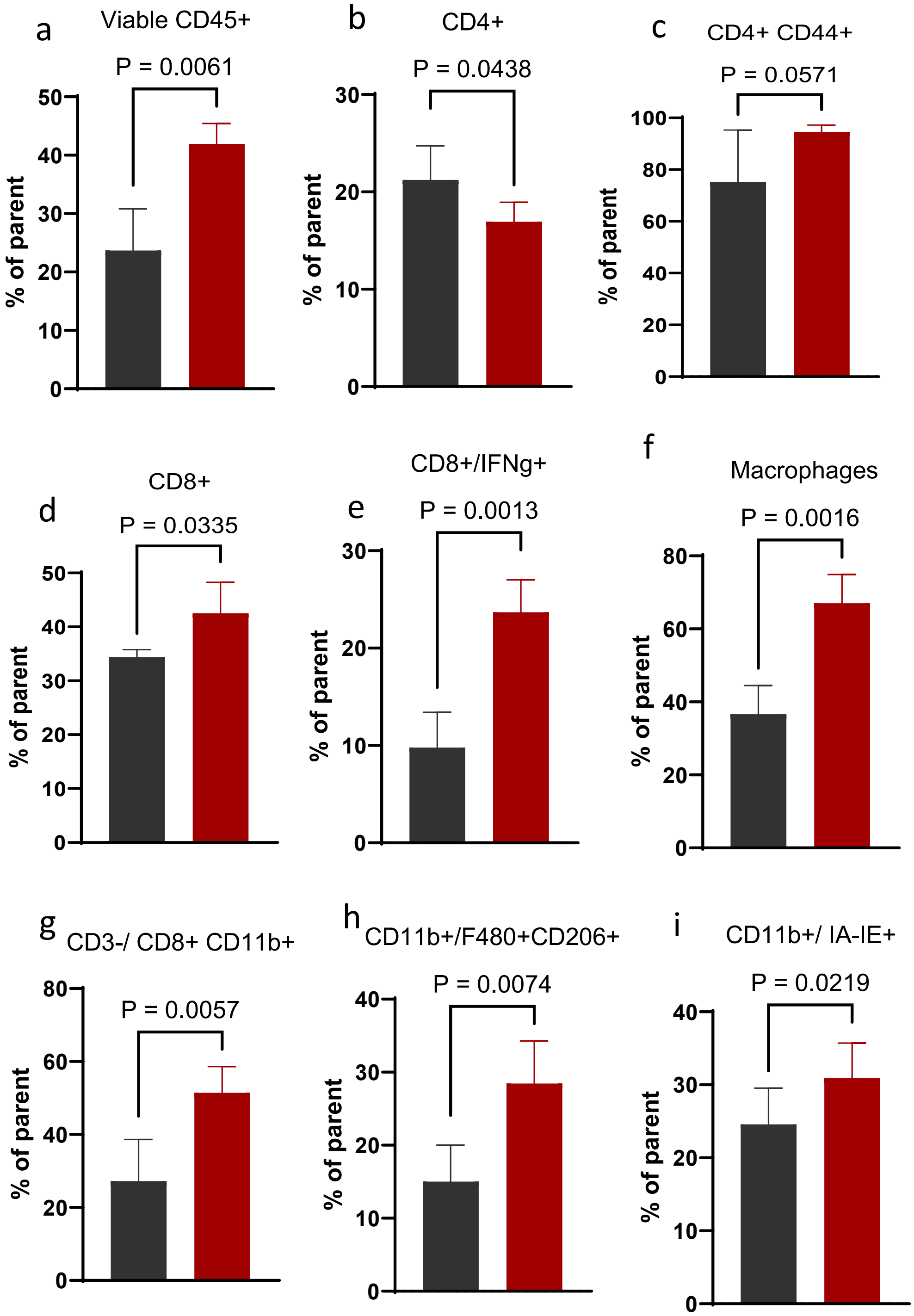

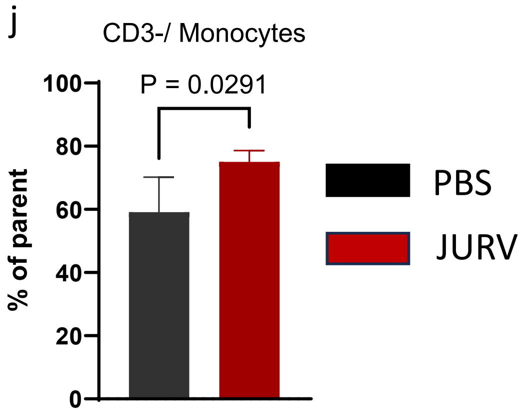

Figure S5. . Analysis of tumor-infiltrating immune cells following intratumoral injection of oncolytic JURV in murine HCC tumors. Immune profiling of PBS or JURV treated syngeneic HEPA 1-6 mice showing percentage of tumor-infiltrating immune cells, including CD45+ (a), CD4+ (b), CD4+CD44+ (c), CD8+ (d), CD8+IFNg+ (e), Macrophages (f), CD3-/CD8-CD11b+ (g), CD11b+/F4/80+ CD206+ (h), CD11b+/IA-IE+ (i) and CD3-/Monocytes (j) cells. The Bartlett test was used to test homogeneity of variance and normality. If the P value of the Bartlett test was no less than P=0.05, ANOVA and a two-sample t test were used to compare group means. If the P value of the Bartlett test was less than 0.05, the Kruskal-Wallis and Wilcoxon rank-sum tests were used to compare group means. The figures demonstrate the potential significant difference of the gated subsets in the CD45+ population, determined by the P value.

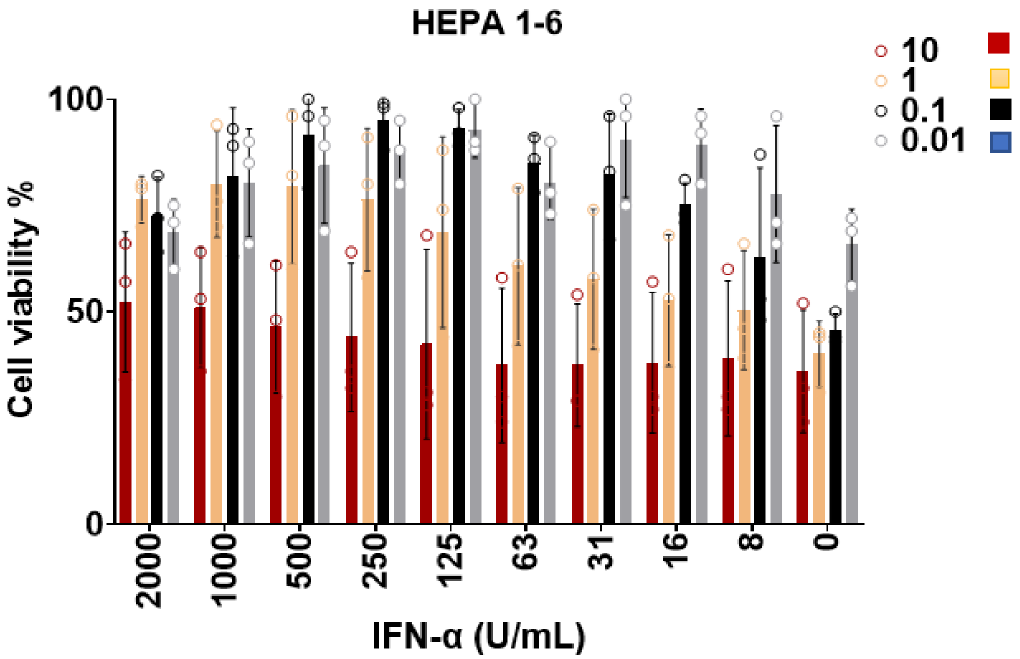

Figure S6. Dose-dependent response to treatment with type interferon on the oncolytic activity of JURV**.** Murine (HEPA 1-6) HCC cells (2.0 x104) were pretreated with various concentrations of universal type I interferon-alpha (IFN-α), infected with viruses at MOI of 10, 1, 0.1 and 0.01. Cell viability was measured using an MTS assay after 48 hours post-infection. Results are from 3 independent experiments and are plotted as mean ± SEM.

Table S1 . List of antibodies for flow cytometry

Markers	Fluorochrome	Clone	Catalog	Isotypes	Vender
**CD45**	FITC	30-F11	553079	Rat IgG2b, κ	BD
**CD3**	BUV395	145-2C11	563565	Armenian Hamster IgG1, κ	BD
**CD4**	BUV737	GK1.5	612761	Rat IgG2b, κ	BD
**CD8**	Percp-Cy5.5	53-6.7	45-0081-82	Rat IgG2a, k	eBioscience
**CD44**	BV711	IM7	103057	Rat IgG2b, k	Biolegend
**CD335**	PE/DAzzle594	29A1.4	137630	Rat IgG2a, κ	Biolegend
**PD-1**	PE	J43	551892	Armenian Hamster IgG2, κ	BD
**Ki67***	BV605	16A8	652413	Rat IgG2a, κ	Biolegend
**Grazyme B***	APC	APC	QA18A28	396408	Biolegend
**IFN-γ***	BV421	XMG1.2	563376	Rat IgG1, κ	BD
**CD11b**	PE-Cy7	M1/70	101216	Rat IgG2b, κ	Biolegend
**F4/80**	BV510	BM8	123135	Rat IgG2a, k	Biolegend
**CD206**	AF700	C068C2	141734	Rat IgG2a, κ	Biolegend
**I-A/I-E**	BV786	2G9	743875	Rat IgG2a, κ	BD
**L/D**	efluo780	NA	65-0865-18	NA	eBioscience

* Ki67, Granzyme B and IFN-γ* are the intracellular targets.
